# Sustained effectiveness of weekly iron-folic acid supplementation and regular deworming over 6 years in women in rural Vietnam

**DOI:** 10.1371/journal.pntd.0005446

**Published:** 2017-04-13

**Authors:** Gerard J. Casey, Ta T. Tinh, Nong T. Tien, Sarah Hanieh, Luca T. Cavalli-Sforza, Antonio Montresor, Beverley-Ann Biggs

**Affiliations:** 1 University of Melbourne, Department of Medicine/RMH at the Doherty Institute, Melbourne, Victoria, Australia; 2 National Institute of Malariology, Parasitology and Entomology, Hanoi, Vietnam; 3 Public Health Nutrition Consultant, previously with World Health Organization, Western Pacific Regional Office, Manila, Philippines; 4 World Health Organization, Geneva, Switzerland; 5 Victorian Infectious Diseases Service, The Royal Melbourne Hospital, Melbourne, Victoria, Australia; Brown University, UNITED STATES

## Abstract

**Background:**

Weekly iron-folic acid (IFA) supplementation and regular deworming is effective for the prevention of iron deficiency and anaemia in women of child-bearing age. Between 2006 and 2013, a program of weekly IFA and biannual deworming was implemented in Yen Bai province, Vietnam. In this study we aimed to determine the effectiveness of the program in reducing anaemia and the prevalence of hookworm infection after 72 months (six years).

**Methods:**

This prospective cohort study followed up a cohort of 389 women of child-bearing age from baseline until six years after the introduction of the weekly IFA (one tablet containing 200 mg ferrous sulphate, 0.4mg folic acid) and deworming (one 400mg tablet of albendazole given twice yearly) program (May 2006 to 2012). In each of the six surveys (baseline and five follow-up surveys) we measured haemoglobin and ferritin, and the burden of soil transmitted helminth (STH) infections, and in the 72 month survey we also administered a questionnaire to assess adherence and possible impediments to participating in the program.

**Results:**

Two hundred and fifty six (65.8%) of the original 389 women enrolled in the cohort attended the final 72 month survey. Haemoglobin levels were 122 g/L [95% C.I. 120, 124] at baseline and increased to 135g/L [95% C.I. 133, 138] after 72 months. The prevalence of anaemia was 37.8% [95% C.I. 31.0, 44.7] at baseline and reduced to 14.3% [95% C.I. 9.5, 19.1]. Hookworm infection prevalence, 75.9% [95% C.I. 68.1, 83.8] at baseline, reduced to 10.2% [95% C.I. 5.4, 15.0] with no moderate or heavy intensity infections. Seventy-two percent of participants reported still taking at least 75% of the weekly supplements, and 85.0% had taken the most recent deworming treatment.

**Discussion:**

Anaemia rates fell significantly during the six-year program, and STH infections were eliminated as a public health risk. Adherence was well maintained but long-term sustainability is challenging in the absence of ongoing external support.

## Author summary

Weekly iron-folic acid (IFA) supplementation combined with regular deworming for women of child bearing age is effective in the prevention of iron deficiency and anaemia. Following a baseline survey, a weekly IFA and regular deworming project was implemented in Yen Bai province, Vietnam in 2006, and after 12 months expanded to the entire province. Haematological parameters, soil transmitted helminth (STH) burden and adherence to the program were monitored periodically until 2012. We found anaemia prevalence fell from 37.8% to 14.3% during the six-year period, and haemoglobin levels increased from 122 g/L to 135g/L. STH infections were essentially eliminated as a public health risk. Seventy-two percent of participants continued to take at least 75% of the weekly supplements, and 85.0% took the most recent deworming treatment. These results show that prevention of anaemia in women of child-bearing age with weekly IFA and regular deworming is feasible and effective over a prolonged period. However, long-term sustainability may be a major challenge in some settings in the absence of ongoing external support.

## Introduction

Anaemia (haemoglobin < 120g/L) is estimated to affect 29.0% of non-pregnant women world-wide. [1] Iron deficiency anaemia (IDA) is the most common form of anaemia in many resource-poor settings. Nutritional anaemia can also be caused by vitamin deficiencies (A, B2, B6, folate, B12, C, E) and deficiencies of other minerals—such as copper, zinc, and in some cases selenium; and severe protein-energy malnutrition.[2] Intestinal infections causing diarrhoea, malabsorption and blood loss (especially hookworm infection) may cause depleted body iron stores and worsen the risk of other micronutrient deficiencies by interfering with digestion and absorption (e.g., vitamin A).[3]

The consequences of IDA are most evident in women of child-bearing age and children. In pregnancy IDA has been linked to premature delivery, higher maternal morbidity, and infants with low birth weight (LBW), lower iron stores and higher anaemia rates. [4, 5] Iron deficiency anaemia in infants and young children may lead to impaired development [4] with long term health implications.[6, 7]

Weekly iron-folic acid (IFA) supplementation is now recommended by WHO for nonpregnant women of child-bearing age living in areas with a prevalence of anaemia above 20% [8, 9], with the aim of improving a women’s iron and folate status prior to conception. The rationale for using intermittent rather than daily supplemental iron relates to the adverse impact of high levels of luminal iron in the gut and the concept that taking iron less frequently may facilitate absorption of iron by mucosal cells and lead to fewer side-effects [10–12]. WHO also recommends that preventive chemotherapy for soil transmitted helminths (STH) be considered in this group in endemic areas where the prevalence is 20% or higher. [13]

However, it is not clear that compliance and effectiveness will be maintained over many years. Long-term sustainability is also of concern, especially when program implementation is dependent on external donor funding. Although the cost of weekly IFA supplementation per woman is estimated to be as little as USD 0.76 cents per year, this still equates to $76,000.00 per 100,000 women per year, a cost few local health administrations can afford. [14]

Between 2006 and 2013, we initiated and supported a program of weekly IFA supplementation, with regular deworming, to a target population of approximately 250,000 women of child-bearing age in Yen Bai Province, Vietnam.[15–17] Our objectives in this study were to document the adherence and effectiveness of weekly IFA supplementation and deworming on rates of hookworm infection, anaemia, and iron deficiency in a cohort of participants followed from baseline to 72 months (6 years) post intervention. We also sought to identify administrative challenges and barriers to ongoing sustainability.

## Methods

### Sampling and the intervention

The intervention has been described previously.[18] Briefly, a demonstration project of weekly IFA (one 200mg tablet of Ferrous Sulphate—equivalent to 60mg elemental iron, 0.4mg folic acid) supplementation combined with four monthly deworming (Albendazole 400mg) for non-pregnant women of child bearing age was initiated in May 2006 in two districts in Yen Bai province over a period of 12 months. Tran Yen and Yen Binh districts were selected for the intervention, being easy to reach, and with both Kinh and ethnic minority populations. All nonpregnant women of reproductive age between 16 and 45 years were eligible for the intervention. Supplements were supplied free of charge. Village health workers (VHWs) were central to the delivery of the intervention to women in their communities, and worked closely with the Women’s Union to mobilize the target population.

A baseline survey was undertaken in November 2005 in a randomly selected cohort of women who were then followed up at 3 and 12 months post introduction of the intervention. [19] Sample selection for the cohort used a stratified multi-stage cluster design, in which 'probability proportional to size' random sampling was used to select primary sampling units (villages) within each district, and secondary sampling units (women) were selected randomly from each village using provincial lists. A sample size of at least 280 was required in the baseline and follow-up surveys to detect an increase in haemoglobin of 5 g/L with a power of 0.9, a type 1 error of .05, and accounting for clustering with an intraclass correlation of 0.2. This number was also sufficient to detect a reduction in hookworm prevalence of 30%. There was no control group as it was considered unethical to actively withhold IFA supplementation and deworming treatment from this group over several years.

### Expansion and implementation of a province-wide weekly IFA/deworming program

After 12 months of the demonstration project, improvements were seen in all measured indices of women’s health: haemoglobin, serum ferritin, anaemia (Hb < 120 g/L), iron deficiency (serum ferritin < 15 mcg/L) and STH prevalence and intensity of infection. [15] As a result, the provincial authorities supported the expansion of the intervention to target reproductive-aged women in all districts of the province (some 250,000 women). The community-based program was administered from the Yen Bai Centre for Malaria Control, through the District Centres for Preventive Medicine to Commune Health Stations and VHWs. The expanded program consisted of a weekly IFA tablet (200mg Ferrous Sulfate) and one tablet of Albendazole (400mg) given twice a year. Eligible women were encouraged to collect their supplements monthly from their VHW, who recorded the woman's name and date of distribution and advised about side-effects and safe storage of supplements. Albendazole was given as observed treatment on locally designated days either at the commune health station or supervised in the village by a commune health worker. National oversight of the program and support for training and educational material development and production was provided by the National Institute of Malariology, Parasitology and Entomology, which also has responsibility for the national hookworm control program. The Provincial Health Department provided salary support for distribution through the health system. WHO donated albendazole tablets but external donor funding was required for the IFA supplements, training and training materials and educational and promotional materials. Thus, the program was not fully supported by the national health system, remaining partly dependent on external financial and administrative support.

### Monitoring and evaluation of the expanded program

The same cohort of women who participated in the baseline, three and 12 month surveys during the demonstration project (as described above) were invited to participate in the 30 month, 54 month, and 72 month follow-up surveys if they were still resident in the same villages.

In all surveys participants were asked to provide a venous blood sample and a faecal sample. Haemoglobin was measured by HemoCue 201+ (HemoCue AB, Angelholm, Sweden) and the STH burden was determined using the Kato-Katz technique as described by Ash et.al. [20] Classification of helminth infection intensity (eggs per gram, EPG) was according to WHO cut offs: 1) Hookworm infections: light = 1–1999, moderate = 2000–3999, heavy ≥ 4000 2) *Ascaris lumbricoides*: light = 1–4999, moderate = 5000–49999, heavy ≥ 50000, and 3) *Trichuris trichiura* light = 1–999, moderate = 1000–9999, heavy ≥ 10000 EPG. For previous surveys, serum ferritin was measured using a sandwich immunoenzymatic assay (IEA; Beckman Coulter Access Reagents, Fullerton, CA). Due to a change in technology at the original laboratory and a subsequent change in the laboratory used, serum ferritin for the 72 month survey was analysed using a Cobas immunoturbidometric test kit (Roche Diagnostics GmbH, D-68298 Mannheim). The structured interview administered during the 72 month survey sought information about whether women had taken the last deworming treatment and how regularly they had taken IFA supplements during the previous 10 weeks, and reasons for noncompliance. It also asked about other factors that may influence adherence such as access to, or provision of, supplements and direct or indirect costs related to travel or lost work time. Adherence with the supplementation regime was defined as taking at least 75% of the supplements provided by the VHW.

### Statistical analysis

Data of surveys collected before year 6 have been analysed and reported previously. [21] By pooling all available survey data we extended the former semi-cross-sectional, semi longitudinal panel design with data collected as part of the final survey. Data analysis was conducted using Stata/IC 11.2 (College Station, TX). Cross-sectional summary data was obtained via linear (haemoglobin, log transformed ferritin) and logistic (prevalence) regression models that were fitted per survey time point, incorporating clustering at village level (Huber-White Sandwich estimator) to obtain robust standard errors. Ferritin data was right-skewed and therefore log transformed to normalise the data. Arithmetic mean haemoglobin, geometric mean ferritin, and prevalence (anaemia, iron deficiency, IDA, hookworm, ascaris, trichuris, and total STH infection) with 95% confidence intervals are presented. At each survey time point and for every outcome, the analysis sample consisted of the available outcome data of all eligible women. Data from women who became pregnant during the study were not excluded from the analysis sample. Longitudinal repeated measures mixed linear and logistic regression was used to investigate the change from baseline to year 6, while accounting for clustering at individual and village level.

Adherence data on weekly IFA supplementation and deworming treatment was summarized descriptively by survey as percentages with 95% confidence intervals. Differences in changes over time in anaemia prevalence were examined by ethnic group within district. The odds ratio of being iron deficient at each post-implementation time point compared to baseline was explored. At 72 months, prevalence of iron deficiency was compared between subgroups formed by district, ethnic group, and gender. Changes in prevalence over time by severity of hookworm, *ascaris*, and *trichuris* infection are presented graphically.

### Ethics

Extensive consultation was undertaken between the project team, communities and community leaders, as well as liaison with village, district and provincial health staff. Village health workers provided participants with information regarding the surveys and written informed consent was documented at the time of enrolment in the surveys. The National Institute of Malariology, Parasitology and Entomology and the Walter and Eliza Hall Institute of Medical Research and Melbourne Health approved of these consent procedures, which were standard NIMPE consent procedures. Potential recruits received a printed plain language statement and oral consent and documented signatures were obtained prior to participation. Participants were informed that they could withdraw from the study at any time and that withdrawal would not affect their routine medical treatment. Participants were all adult women between the ages of sixteen and forty-five; there were no minors included. The project was approved by the Human Research Ethics Committees of the National Institute of Malariology, Parasitology and Entomology (Hanoi, Vietnam), and the Walter and Eliza Hall Institute of Medical Research and Melbourne Health (Melbourne, Australia) and all ethics committees specifically approved the use of participants of reproductive age between sixteen and forty-five years of age.

## Results

**Fig 1:**
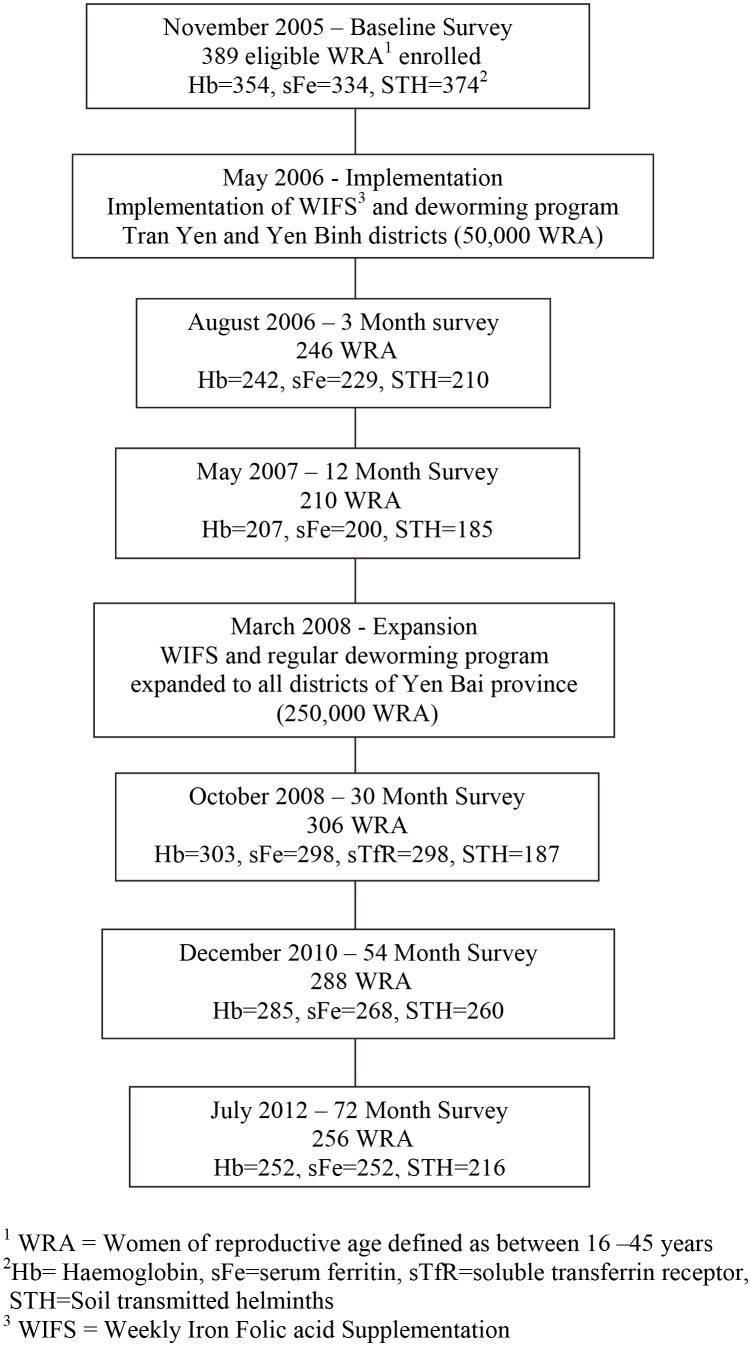
Timeline of surveys and intervention (adapted from [21]). Notes: ^1^ WRA = Women of Reproductive Age defined as between 16–45 years, ^2^Hb = Haemoglobin, sFe = serum Ferritin, sTfR = soluble Transferrin Receptor, STH = STHs, ^3^ WIFS = Weekly Iron Folic acid Supplementation.

The timeline and participation rates for this and previous surveys are shown in Fig 1. Of the 389 women originally surveyed in 2005, 256 returned for the survey in 2012. Two hundred and fifty two women provided blood samples, 216 provided stool samples and six women left before completing the interview. One hundred and seventy eight women (72.0% [95% C.I. 63.6%, 80.4%]) were still taking at least 75% of the supplements they received. A further 29 women received the supplements but either only took them sometimes or gave them to someone else. The forty-three remaining women did not receive the supplements. These latter women were concentrated in six communes in Tran Yen district. Deworming treatment was received by 85.0% (95% C.I.79.5, 90.5) of women (212/249) during the last campaign but two of them did not take it. The adherence over time with taking IFA supplements and deworming treatment is shown in Table 1.

**Table 1:** Adherence^1^ with weekly IFA supplementation and deworming treatment over time^2^.

	3m survey Adherence		12m survey Adherence		30m survey Adherence		54m survey Adherence		72m survey Adherence	
	N	Per cent (95% C.I.)	N	Per cent (95% C.I.)	N	Per cent (95% C.I.)	N	Per cent (95% C.I.)	N	Per cent (95% C.I.)
Weekly IFA supplementation	243	97.9 (95.8, 100)	204	98.5 (96.8, 100)	305	93.1 (87.3, 98.9)	285	76.1 (67.9, 84.4)	250	72.0 (63.6, 80.4)
Deworming treatment	246	89.0 (85.1, 92.9)	210	88.6(81.7, 95.5)	306	87.9 (80.1, 95.7)	288	95.5 (92.8, 98.1)	247	85.0 (79.5, 90.5)

Notes:

^1^ Defined as the percentage of women taking at least 75% of the supplements provided.

^2^ No women were taking supplements at baseline.

The change in mean haemoglobin and ferritin, and the prevalence of anaemia, iron deficiency anaemia and moderate/heavy STH infection over the 72 month period of program implementation are shown in Table 2. By 2012 the overall mean haemoglobin level was 135g/L [95% C.I. 133, 138]. Based on the mixed-effects model this represents an increase from baseline of 13g/L [95% C.I. 11, 15]. The prevalence of anaemia reduced from 38% [95% C.I. 31, 45] to 14% [95%C.I. 9, 19]. Iron deficiency anaemia reduced from 14% [95%C.I. 10, 19] to 3.9% [95% C.I. 2, 6].

**Table 2:** Mean haemoglobin, geometric mean ferritin and prevalence of anaemia, iron deficiency and iron deficiency anaemia, and prevalence of STH infections by WHO categories over time. [32]

	Baseline Prevalence		3 month survey Prevalence		12 month survey Prevalence		30 month survey Prevalence		54 month survey Prevalence		72 month survey Prevalence	
	N	Mean (95% C.I.)	N	Mean (95% C.I.)	N	Mean (95% C.I.)	N	Mean (95% C.I.)	N	Mean (95% C.I.)	N	Mean (95% C.I.)
Haemoglobin (g/L)	354^1^	122 (120, 125)	242	126 (124, 128)	207	130 (128, 132)	303	130 (128, 132)	285	131 (128, 134)	252	135 (133, 138)
Ferritin (mcg/L)	334^1^	28 (23.9, 32.7)	229	37.4 (32.5, 43.0)	200	47.6 (41.9, 54.2)	298	52.4 (45.1, 60.8)	268	53.9 (46.5, 62.4)	252	47.1 (38.8, 57.3)
Anaemia	354	37.8 (31.0, 44.7)	242	26.4 (19.7, 33.2)	207	19.3 (13.2, 25.4)	303	18.8 (13.8, 23.8)	285	17.9 (12.5, 23.3)	252	14.3 (9.5, 19.1)
Iron deficiency	334	25.0 (18.5, 27.6)	229	13.1 (8.7, 17.5)	200	7.0 (3.4, 10.5)	298	9.1 (5.8, 12.3)	268	8.2 (4.9, 11.5)	252	17.1 (12.4, 21.7)
IDA	334	14.4 (9.6, 19.1)	229	6.1 (3.6, 8.7)	200	2.0 (0.1, 3.9)	297	3.7 (1.3, 6.1)	268	3.0 (0.6, 5.4)	252	4.0 (1.5, 6.4)
Hookworm	374^1^		210		185		187		260		216	
- light infection		59.6(54.6, 64.6)		52.9 (46.1 59.6)		27.6 (21.1, 34.0)		11.8 (7.1, 16.4)		11.5 (7.6, 15.4)		10.1 (6.1, 14.2)
- Mod/Heavy		16.3 (10.1, 12.6)		4.3, (1.5, 7.0)		2.1 (1.1, 4.3)		10.0, (4.0, 16.3)		0		0
*Ascaris*	374		210		185		187		260		216	
- light infection		12.0 (8.7, 15.3)		5.2 (2.2, 8.3)		3.8 (1.0, 6.5)		2.7 (0.4, 5.0)		4.6 (0.2, 7.2)		1.4 (0.0, 2.9)
- Mod/heavy		7.5, (4.8, 10.2)		1.4, (0, 3.0)		0.5, (0, 1.6)		1.6, (0, 3.4)		0		1.0 (0, 2.2)
*Trichuris*	374		210		185		187		260		216	
- Light infection		27.3 (22.7, 31.8)		21.9 (16.3, 27.5)		11.4 (6.8, 15.9)		6.4 (2.9, 9.9)		2.7 (0.7, 4.7)		2.3 (0.3, 4.3)
- Mod/Heavy		2.1, (0.7, 3.6)		0		0		3.2, (0.7, 5.8)		0		0
Total STH infection	374	83.7 (79.0, 87.4)	210	67.1 (60.8, 73.5)	185	35.7 (28.7, 42.6)	187	28.3 (21.9, 34.8)	260	16.5, (12.0, 21.1)	216	13.9 (9.3, 18.5)

^1^. Of the 389 eligible women, the proportion of women with haemoglobin measurements at all survey time points was 26.5%. The proportion with five, four, three, two, one, or no haemoglobin measurement (s) was 22.9%, 20.0%, 14.7%, 9.5%, 5.9%, and 0.5%, respectively. A similar missing data pattern was observed for ferritin. For hookworm, the proportion of eligible women with data on hookworms at all survey time points was 17.7%. The proportion with five, four, three, two, one, or no data on hookworms was 18.8%, 16.7%, 19.8%, 15.7%, 10.3%, and 1.0% respectively. Pregnant women were included in the analysis.

**Fig 2:**
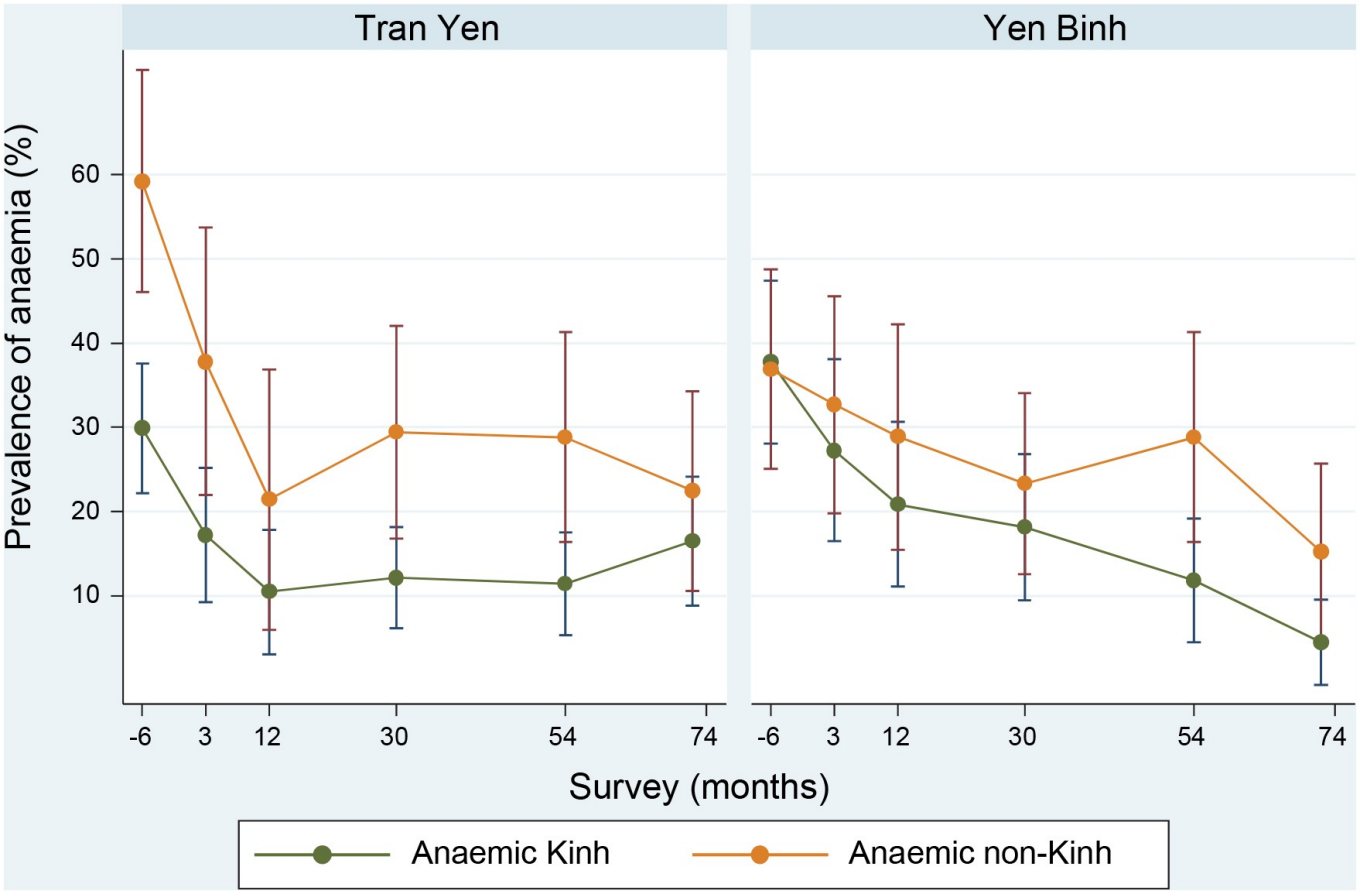
Change in anaemia prevalence by ethnic grouping (Kinh or non-Kinh) and district.

**Fig 3:**
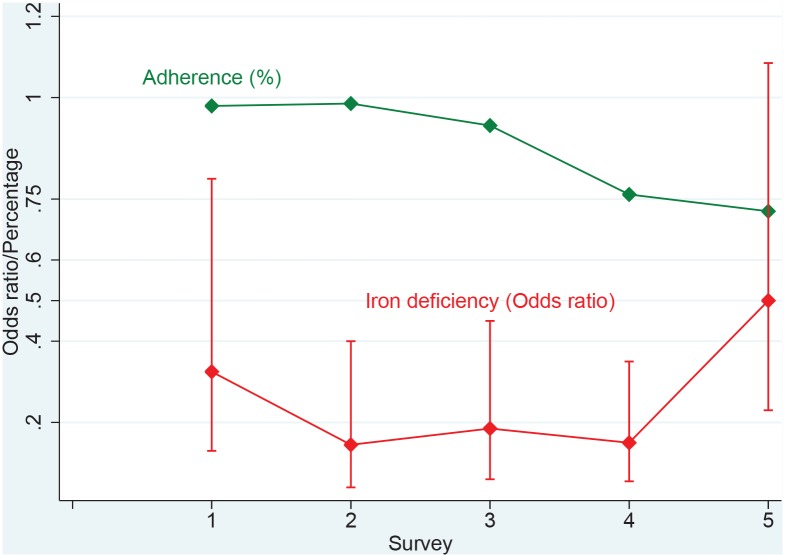
Odds ratios for iron deficiency compared to baseline and prevalence of adherence to taking iron tablets.

The relative change in anaemia over time by district and ethnic group of Kinh or non-Kinh is shown in Fig 2. The anaemia prevalence dropped significantly from previous levels in all population groups, in the first 12 months of the intervention. In the following years anaemia prevalence continued to decrease significantly, in both the Kinh and non-Kinh ethnic groups, in Yen Binh district. However in Tran Yen district it remained static in the following 5 years, between 11 and 17% for the Kinh, and between 21 and 29% for the non-Kinh. The prevalence of iron deficiency in the 72 month survey was 43/252 (17%, [95%C.I. 10, 24]). This was an increase on previous levels and corresponded to falling adherence rate (Fig 3). There was a higher prevalence of iron deficiency in Tran Yen, 30/140 (21%, [95% C.I. 15, 28]) compared to Yen Binh, 13/112 (12%, [95%C.I. 6, 18]). Iron supplements were reportedly taken by 25/29 (86%, [95%C.I. 73, 100]) of iron deficient women in Tran Yen, and 9/13 (69%, [95% C.I. 40, 98]) in Yen Binh district. Iron deficiency was more prevalent among women from ethnic minority groups (non-Kinh) 23/95 (24%, [95% C.I. 16, 33]) than Kinh women, 20/157 (13%, [95%C.I. 7, 18]). The main reason for not taking supplements was unavailability, but some women also reported not needing them because they felt well.

**Fig 4:**
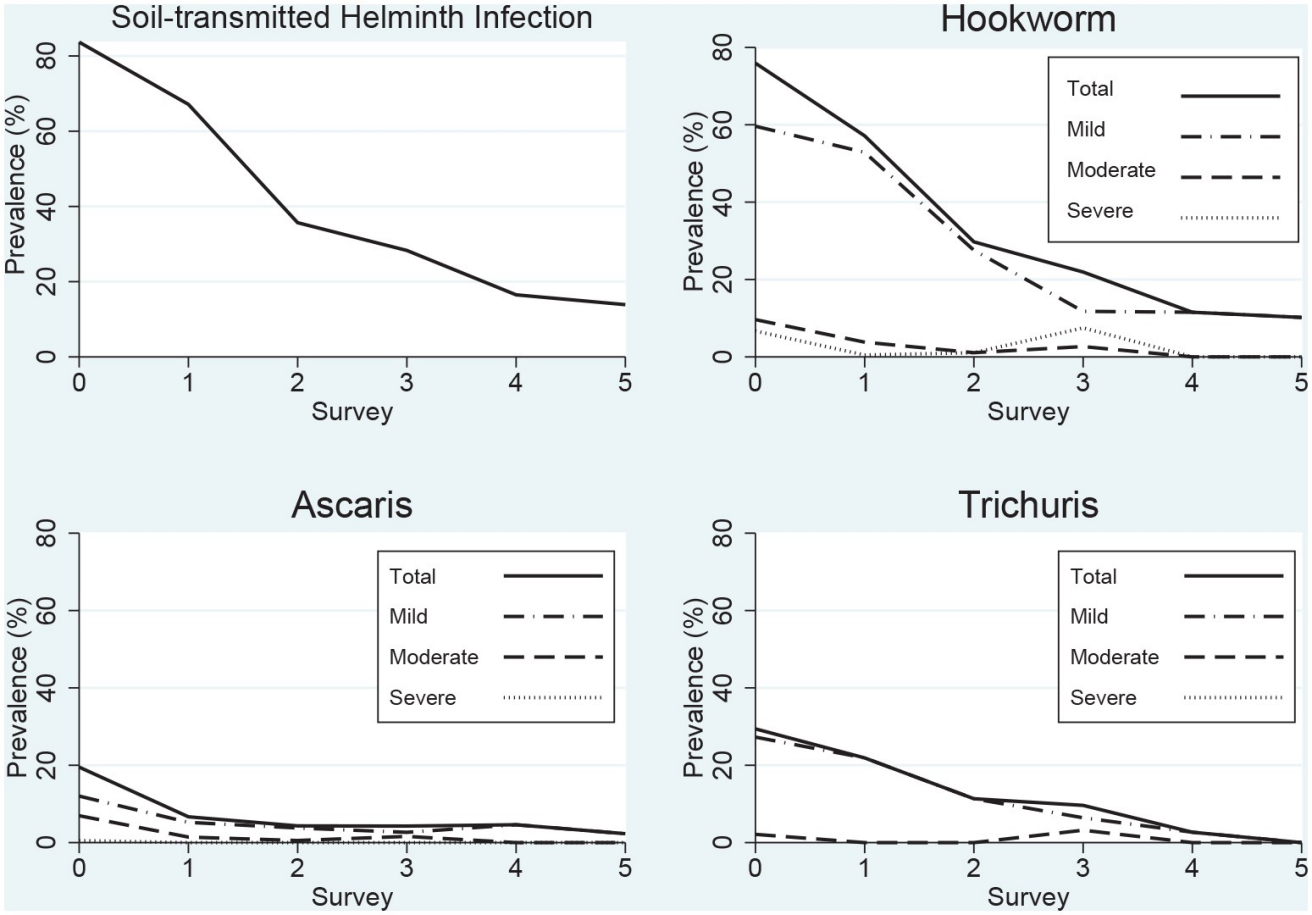
Change in Soil Transmitted Helminth (STH) burden during the implementation of the WIFS/deworming program (2006–2012).

The overall prevalence of STH infection fell from 83.7% [95% C.I. 77.2, 90.2] to 13.9% [95% C.I. 8.7, 19.1], and hookworm infection from 75.9% [95% C.I. 68.1, 83.8] to 10.2% [95% C.I. 5.4, 15.0], while moderate and heavy infections were essentially eliminated (Fig 4).

## Discussion

We report the effectiveness of a community-wide weekly IFA supplementation and regular deworming program in a population of non-pregnant rural Vietnamese women after 72 months. It is one of the few studies to evaluate an *intermittent* iron supplementation and deworming program for nonpregnant women over a period of many years and offers unique insights into the effectiveness and sustainability of the WHO-recommended approach to prevention of iron-deficiency anaemia in this population. Haemoglobin levels remained well above the baseline mean and the prevalence of anaemia continued to fall in most sections of the population. Moderate and heavy intensity STH infections were virtually eliminated and only 10% of women still had light infections (mostly hookworm).

In 2011, WHO recommended weekly IFA supplementation for non-pregnant women of reproductive age in populations with an anaemia prevalence of 20% or higher, given in three month cycles with a three month gap between each cycle. [8] In the Yen Bai setting, it took 12 months of weekly supplementation for the population prevalence of anaemia to drop below 20% and up to 6 years (72 months) for the prevalence to be significantly below the 20% level. This slow rate of decline in anaemia levels has also been noted in other studies [22, 23] and suggests greater benefits if weekly or biweekly supplementation is given continuously, at least in the first year, and continued for several years.

The increased risk of iron deficiency in women of child bearing age is often compounded by hookworm infection in endemic areas. WHO has previously recommended that this group be included in preventive chemotherapy programs for STH (except during the first trimester of pregnancy), and suggest achieving synergy by packaging this intervention with other interventions. [13] These guidelines are currently being updated and will provide more detail to assist program managers considering this intervention. [24]

We observed reduced but still reasonable adherence with weekly IFA after 54 and 72 months (76% and 72% respectively), suggesting that the program remained popular with the target population. However, despite the relatively high reported adherence rates in Tran Yen district we noted a rise in the prevalence of iron deficiency since the 54 month survey. We found that the supply of IFA supplements had been interrupted in certain communes in Tran Yen district during this time, which may explain the rise in iron deficiency in this district. The failure to achieve a consistent supply of supplements in these communes may be due to inadequate training of new health staff over the 6 years of implementation as there was considerable turnover of commune staff and VHWs (GC personal communication). It is important to note that STH infections of moderate and heavy intensity for any STH species remained at or less than 1%. As these heavier worm burdens infections are main cause of morbidity, we conclude that the deworming intervention virtually eliminated all STH-related morbidity.

Limitations of the study included a reduced participation rate in the later surveys, in spite of the efforts of local village and commune health workers publicising the surveys. This was most likely due to the long follow-up period, and the movement of some families out of the area. The relatively high loss to follow-up may have biased our estimates of adherence and effectiveness, as non-adherent women may have chosen not to take part, while healthier adherent women may have remained engaged. As well, women who were feeling tired, or had illness, and those with poorer economic circumstances may have been less likely to adhere and/or to attend for surveys. This would have resulted in a healthier cohort at the end of the program, making the intervention appear more effective. Other limitations were that adherence data relied on self-reporting rather than objective documentation, and women who attended the survey may have exaggerated their adherence. Ferritin levels were measured using different methodology in this, compared to previous surveys. However both were commercial assays and we have no reason to believe that this change contributed to the lower mean ferritin observed in this survey. We did not include a control group as the research team and provincial authorities felt this would be unethical for a long term program. However, we are unaware of significant improvements in economic conditions during the six year period that may have accounted for the results presented here. Indeed, the global financial crisis commenced soon after program expansion and so deterioration in community living standards may have been expected. Improvements in main road infrastructure did occur but we did not observe a change in living conditions at village level (Casey, personal communication). The project was conducted in a remote rural region of Vietnam and may not be generalizable to other areas or ethnic groups where the prevalence and causes of anaemia may differ.

The sustainability of weekly IFA/deworming programs for the large populations for whom they are recommended is country-specific. The program in Yen Bai province was mainly externally funded, and so was never fully incorporated as a national or provincially-funded program. In post program debriefings, provincial health and finance officials emphasised that, while the program was well accepted by the population and effective and cheap on a per person basis (0.76USD/woman/year), the cost of supplying weekly supplements to the target population (approx. $200,000 per annum) was beyond the capacity of the province’s health budget. While they were prepared to cover the human resource distribution costs, they were not able to support purchase of IFA supplements, development and production of educational materials, and training (G Casey, T Tinh, personal communication). Multiple micronutrient supplementation is even more expensive, even though it may be indicated in settings with higher rates of micronutrient deficiencies. [25]

There are however encouraging signs for the long-term sustainability of community-based WIFS/deworming programs in some other countries, especially India.[17, 26] Based on sound evidence from the field, [23, 27] the Indian Government has produced an operational framework for universal weekly IFA supplementation for adolescents in school and adolescent females not attending school. [28] Responsibility for the national program, from policy formulation and resource allocation to monitoring and review, has been allocated to the Ministry of Health and Family Welfare [29]. Given that this program is projected to cover 130 million adolescents, it may encourage more countries with at-risk populations to provide resources for similar national programs. Likewise, in Cambodia, the use of weekly iron and folic acid supplementation has been progressively extended, over a 10 year period, to cover most schools (LTC-S personal communication).

A revolving fund approach can help sustain the program, by selling the supplements. This approach was successfully used in the weekly IFA supplementation programme of Hai Duong province, Viet Nam, where the supplements were sold to non-pregnant women through the Women’s Union network and provided free of charge when women were pregnant, according to the Vietnamese health policy [30]. Over a year, a non-pregnant woman would spend the equivalent of US$0.96, which was acceptable for rural women in Vietnam. Funds gained from the sales of the supplements were used to pay for an incentive for Women’s Union collaborators to sell the supplement (20%), and for management costs and regular communication and promotion activities in the communes (30%). The remaining 50% was held in a local bank under the supervision of a district steering committee, and used to purchase new supplements, to continue the programme beyond its initial financing period. [30]

A review of weekly IFA supplementation programs conducted in Cambodia, The Philippines and Viet Nam concluded that women are willing and able to purchase supplements when they are widely available and affordable, including in poor rural areas and schools. [31] In the Cambodian factories, where supplements had to be provided free of charge because local laws forbade their sale, WRA asked that the supplements be sold outside the factories so that they could continue taking them in the future. In each country, the programme’s success led to expanding weekly iron-folic acid supplementation through larger-scale programmes. The variety of social marketing and community mobilization strategies used in the three countries in schools, factories, and communities (discussed in the above mentioned programme review) provide valuable lessons for replicating this approach in other countries. [31]

An analysis of 10 weekly IFA programmes for the prevention and control of anaemia in women, which took place in 6 countries, confirmed that high compliance in taking the supplements can be achieved, irrespective of supervision, provided recipients are convinced of the benefits through an effective communication strategy, with the participation of several stakeholders, and a system in place for monitoring consumption.[17]

In conclusion, the program of free weekly IFA supplementation and regular deworming for women of reproductive age in Yen Bai province ran successfully for 6 years with external inputs of supplements, training and education. It was well received by the population, with good adherence, and resulted in major reductions in anaemia and STH infection. Sustainability will probably require full integration into Vietnam’s national health system. A complementary approach to be considered, successfully used both elsewhere in Viet Nam and in other countries, is to sell the supplements at an affordable price while promoting them through social marketing, thus creating and maintaining demand for the product.

## Supporting information

S1 Checklist Strobe checklist. (DOC)
